# Atrial fibrillation classification based on convolutional neural networks

**DOI:** 10.1186/s12911-019-0946-1

**Published:** 2019-10-29

**Authors:** Kwang-Sig Lee, Sunghoon Jung, Yeongjoon Gil, Ho Sung Son

**Affiliations:** 1 AI Center, Korea University College of Medicine, Seoul, South Korea; 2 HUINNO Co., Ltd., Seoul, South Korea; 3 Department of Thoracic and Cardiovascular Surgery, Korea University College of Medicine, 73 Inchon-ro, Seongbook-gu, Seoul, 02841 South Korea

**Keywords:** Atrial fibrillation, Convolutional neural networks, Alex networks, Residual networks

## Abstract

**Background:**

The global age-adjusted mortality rate related to atrial fibrillation (AF) registered a rapid growth in the last four decades, i.e., from 0.8 to 1.6 and 0.9 to 1.7 per 100,000 for men and women during 1990–2010, respectively. In this context, this study uses convolutional neural networks for classifying (diagnosing) AF, employing electrocardiogram data in a general hospital.

**Methods:**

Data came from Anam Hospital in Seoul, Korea, with 20,000 unique patients (10,000 normal sinus rhythm and 10,000 AF). 30 convolutional neural networks were applied and compared for the diagnosis of the normal sinus rhythm vs. AF condition: 6 Alex networks with 5 convolutional layers, 3 fully connected layers and the number of kernels changing from 3 to 256; and 24 residual networks with the number of residuals blocks (or kernels) varying from 8 to 2 (or 64 to 2).

**Results:**

In terms of the accuracy, the best Alex network was one with 24 initial kernels (i.e., kernels in the first layer), 5,268,818 parameters and the training time of 89 s (0.997), while the best residual network was one with 6 residual blocks, 32 initial kernels, 248,418 parameters and the training time of 253 s (0.999). In general, the performance of the residual network improved as the number of its residual blocks (its depth) increased.

**Conclusion:**

For AF diagnosis, the residual network might be a good model with higher accuracy and fewer parameters than its Alex-network counterparts.

## Background

Heart disease is the leading cause of disease burden in the world and Korea [1–6]. Cardiovascular disease accounted for the greatest part of global mortality in Year 2013 (Y2013 hereafter), i.e., 32% (17 million) of 54 million deaths in the world [1]. The global age-adjusted mortality rate per 100,000 related to atrial fibrillation (AF), the most common form of irregular heartbeat, registered a rapid growth from 0.8 to 1.6 (or 0.9 to 1.7) for men (or women) during 1990–2010 [2]. This global pattern is consistent with its local counterpart in Korea. Heart disease was the second cause of death in Korea for Y2016 (58.2 per 100,000) [3] and the third cause of disease burden in the nation for Y2010 (562 disease-adjusted life years per 100,000) [4]. Indeed, hospitalization for AF in the nation increased by 420% from 767 to 3986 per 1 million from Y2006 to Y2015 [5].

In the context above, an increasing amount of research has used deep neural networks to classify (diagnose) AF and other types of arrhythmia, given their superior performance compared to other machine learning methods [6–12]. This line of research applied convolutional neural networks (i.e., Alex, Residual) [6–10], their recurrent counterparts (i.e., long short term memory) [11] or both [12] to achieve the accuracy range of 80–99% with varying numbers of class numbers for electrocardiogram data. Most of these studies employed public data such as Massachusetts Institute of Technology-Beth Israel Hospital (MIT-BIH) arrhythmia data. Also, more comparison might be needed for a variety of deep neural networks with different degrees of their depth (i.e., numbers of their layers) and varying numbers of their kernels for the diagnosis of arrhythmia.

For this reason, this study used electrocardiogram (ECG) data in a general hospital and various convolutional neural networks for diagnosing arrhythmia. ECG is a graph of heartbeat in voltage versus time recorded by electrodes on the chest and the limbs. A normal ECG wave consists of five parts, i.e., P (atrial contraction), Q (downward deflection immediately before ventricular contraction), R (the peak of ventricular contraction), S (downward deflection immediately after ventricular contraction) and T (ventricular recovery) (Additional file 1: Figure S1A). Its AF counterpart shows an irregular pattern, for example, lacking a P part with an irregularly irregular QRS part (Additional file 1: Figure S1B). These ECG waves are arranged in a grid of four columns and three rows, i.e., the first column for “limb leads” (or voltage differences measured by limb electronodes) [I, II, III in Additional file 1: Figure S2], the second column for “augmented limb leads” (or voltage differences measured by limb electronodes with a different combination so called Goldberger’s central terminal) [aVR, aVL, aVF in Additional file 1: Figure S2] and the last two columns for “precordial leads” (or voltage differences measured by chest electronodes) [V1-V6 in Additional file 1: Figure S2]. Based on the ECG data in a general hospital, 30 convolutional neural networks were applied and compared in this study for the diagnosis of the normal sinus rhythm (NSR) vs. AF condition: 6 Alex networks with 5 convolutional layers, 3 fully connected layers and the number of kernels changing from 3 to 256; and 24 residual networks with the number of residuals blocks (or kernels) varying from 8 to 2 (or 64 to 2).

## Methods

Data came from Anam Hospital in Seoul, Korea, with 20,000 unique participants (10,000 NSR and 10,000 AF). Preprocessing processes for the ECG data are shown in Additional file 1: Figures S2A, B and C, i.e., removing the background grid, selecting target signals and getting numerical values, respectively. Here, selecting target signals consists of three sub-processes based on OpenCV functions such as connectedComponents: (1) computing connected components in a binary image with 8-connectivity; (2) computing the bounding rectangle for each connected component; and (3) selecting the component whose bounding rectangle has the longest width. In Tables 1 and 2, input dimensions and the number of kernels are described for 30 convolutional neural networks (i.e., 6 Alex networks and 24 residual networks) for the diagnosis of the NSR vs. AF condition in this study: Alex 1–6 with 5 convolutional layers, 3 fully connected layers and the number of kernels changing from 3 to 256; and Residual 1–1 to Residual 4–6 with the number of residuals blocks (or kernels) varying from 8 to 2 (or 64 to 2). The original Alex network, which consists of 5 convolutional layers and 3 fully connected layers with Rectified Linear Unit (ReLU) activation functions, topped the 2012 ImageNet Large Scale Visual Recognition Challenge and demonstrated its superior performance over its traditional sigmoid activation function counterparts [13]. In convolutional layers of the Alex network, a kernel (or feature detector) slides across input data and operates “convolution”, i.e., calculating the dot product of its elements and their input-data counterparts, detecting specific features of the input data, e.g., the shape of a dog’s ear which differentiates it from a cat.

**Table 1:** Alex-Network Architecture: Input Dimension & Number of Kernels

Layer/Model	Alex 1	Alex 2	Alex 3	Alex 4	Alex 5	Alex 6
Convolution	(1, 500, 96)^a^	(1, 500, 48)	(1, 500, 24)	(1, 500, 12)	(1, 500, 6)	(1, 500, 3)
Pooling	(1, 250, 96)	(1, 250, 48)	(1, 250, 24)	(1, 250, 12)	(1, 250, 6)	(1, 250, 3)
Convolution	(1, 250, 256)	(1, 250, 128)	(1, 250, 64)	(1, 250, 32)	(1, 250, 16)	(1, 250, 8)
Pooling	(1, 125, 256)	(1, 125, 128)	(1, 125, 64)	(1, 125, 32)	(1, 125, 16)	(1, 125, 8)
Convolution	(1, 125, 384)	(1, 125, 192)	(1, 125, 96)	(1, 125, 48)	(1, 125, 24)	(1, 125, 12)
Convolution	(1, 125, 384)	(1, 125, 192)	(1, 125, 96)	(1, 125, 48)	(1, 125, 24)	(1, 125, 12)
Convolution	(1, 125, 256)	(1, 125, 128)	(1, 125, 64)	(1, 125, 32)	(1, 125, 16)	(1, 125, 8)
Pooling	(1, 63, 256)	(1, 63, 128)	(1, 63, 64)	(1, 63, 32)	(1, 63, 16)	(1, 63, 8)
Fully Connected	(1024)	(1024)	(1024)	(1024)	(1024)	(1024)
Fully Connected	(1024)	(1024)	(1024)	(1024)	(1024)	(1024)
Output	(2)	(2)	(2)	(2)	(2)	(2)

^a^(1, 500, 96) Input Dimension 1, Input Dimension 2, Number of Kernels

**Table 2:** Residual-Network Architecture: Input Dimension & Number of Kernels

Layer/Model	Residual 1–1	Residual 1–2	Residual 1–3	Residual 1–4	Residual 1–5	Residual 1–6
Convolution	(1, 1000, 64)^a^	(1, 1000, 32)	(1, 1000, 16)	(1, 1000, 8)	(1, 1000, 4)	(1, 1000, 2)
Pooling	(1, 500, 64)	(1, 500, 32)	(1, 500, 16)	(1, 500, 8)	(1, 500, 4)	(1, 500, 2)
Residual Block	(1, 500, 64)	(1, 500, 32)	(1, 500, 16)	(1, 500, 8)	(1, 500, 4)	(1, 500, 2)
Residual Block	(1, 500, 64)	(1, 500, 32)	(1, 500, 16)	(1, 500, 8)	(1, 500, 4)	(1, 500, 2)
Residual Block	(1, 250, 128)	(1, 250, 64)	(1, 250, 32)	(1, 250, 16)	(1, 250, 8)	(1, 250, 4)
Residual Block	(1, 250, 128)	(1, 250, 64)	(1, 250, 32)	(1, 250, 16)	(1, 250, 8)	(1, 250, 4)
Residual Block	(1, 125, 256)	(1, 125, 128)	(1, 125, 64)	(1, 125, 32)	(1, 125, 16)	(1, 125, 8)
Residual Block	(1, 125, 256)	(1, 125, 128)	(1, 125, 64)	(1, 125, 32)	(1, 125, 16)	(1, 125, 8)
Residual Block	(1, 63, 512)	(1, 63, 256)	(1, 63, 128)	(1, 63, 64)	(1, 63, 32)	(1, 63, 16)
Residual Block	(1, 63, 512)	(1, 63, 256)	(1, 63, 128)	(1, 63, 64)	(1, 63, 32)	(1, 63, 16)
Pooling	(1, 1, 512)	(1, 1, 256)	(1, 1, 128)	(1, 1, 64)	(1, 1, 32)	(1, 1, 16)
Output	(2)	(2)	(2)	(2)	(2)	(2)
Layer/Model	Residual 2–1	Residual 2–2	Residual 2–3	Residual 2–4	Residual 2–5	Residual 2–6
Convolution	(1, 1000, 64)	(1, 1000, 32)	(1, 1000, 16)	(1, 1000, 8)	(1, 1000, 4)	(1, 1000, 2)
Pooling	(1, 500, 64)	(1, 500, 32)	(1, 500, 16)	(1, 500, 8)	(1, 500, 4)	(1, 500, 2)
Residual Block	(1, 500, 64)	(1, 500, 32)	(1, 500, 16)	(1, 500, 8)	(1, 500, 4)	(1, 500, 2)
Residual Block	(1, 500, 64)	(1, 500, 32)	(1, 500, 16)	(1, 500, 8)	(1, 500, 4)	(1, 500, 2)
Residual Block	(1, 250, 128)	(1, 250, 64)	(1, 250, 32)	(1, 250, 16)	(1, 250, 8)	(1, 250, 4)
Residual Block	(1, 250, 128)	(1, 250, 64)	(1, 250, 32)	(1, 250, 16)	(1, 250, 8)	(1, 250, 4)
Residual Block	(1, 125, 256)	(1, 125, 128)	(1, 125, 64)	(1, 125, 32)	(1, 125, 16)	(1, 125, 8)
Residual Block	(1, 125, 256)	(1, 125, 128)	(1, 125, 64)	(1, 125, 32)	(1, 125, 16)	(1, 125, 8)
Pooling	(1, 1, 256)	(1, 1, 128)	(1, 1, 64)	(1, 1, 32)	(1, 1, 16)	(1, 1, 8)
Output	(2)	(2)	(2)	(2)	(2)	(2)
Layer/Model	Residual 3–1	Residual 3–2	Residual 3–3	Residual 3–4	Residual 3–5	Residual 3–6
Convolution	(1, 1000, 64)	(1, 1000, 32)	(1, 1000, 16)	(1, 1000, 8)	(1, 1000, 4)	(1, 1000, 2)
Pooling	(1, 500, 64)	(1, 500, 32)	(1, 500, 16)	(1, 500, 8)	(1, 500, 4)	(1, 500, 2)
Residual Block	(1, 500, 64)	(1, 500, 32)	(1, 500, 16)	(1, 500, 8)	(1, 500, 4)	(1, 500, 2)
Residual Block	(1, 500, 64)	(1, 500, 32)	(1, 500, 16)	(1, 500, 8)	(1, 500, 4)	(1, 500, 2)
Residual Block	(1, 250, 128)	(1, 250, 64)	(1, 250, 32)	(1, 250, 16)	(1, 250, 8)	(1, 250, 4)
Residual Block	(1, 250, 128)	(1, 250, 64)	(1, 250, 32)	(1, 250, 16)	(1, 250, 8)	(1, 250, 4)
Pooling	(1, 1, 128)	(1, 1, 64)	(1, 1, 32)	(1, 1, 16)	(1, 1, 8)	(1, 1, 4)
Output	(2)	(2)	(2)	(2)	(2)	(2)
Layer/Model	Residual 4–1	Residual 4–2	Residual 4–3	Residual 4–4	Residual 4–5	Residual 4–6
Convolution	(1, 1000, 64)	(1, 1000, 32)	(1, 1000, 16)	(1, 1000, 8)	(1, 1000, 4)	(1, 1000, 2)
Pooling	(1, 500, 64)	(1, 500, 32)	(1, 500, 16)	(1, 500, 8)	(1, 500, 4)	(1, 500, 2)
Residual Block	(1, 500, 64)	(1, 500, 32)	(1, 500, 16)	(1, 500, 8)	(1, 500, 4)	(1, 500, 2)
Residual Block	(1, 500, 64)	(1, 500, 32)	(1, 500, 16)	(1, 500, 8)	(1, 500, 4)	(1, 500, 2)
Pooling	(1, 1, 64)	(1, 1, 32)	(1, 1, 16)	(1, 1, 8)	(1, 1, 4)	(1, 1, 2)
Output	(2)	(2)	(2)	(2)	(2)	(2)

^a^(1, 1000, 64), Input Dimension 1, Input Dimension 2, Number of Kernels

Then, many scholars tried to improve the original Alex network by deepening it (or adding more layers to it). However, this attempt turned out to be futile given that it brings back the old problem of gradient vanishing (the gradient of the loss with respect to the weight becomes 0 quickly) [14]. For this reason, several scholars introduced the original residual network with new features of residual mapping and shortcut connection, which managed its considerable depth (e.g., 152 layers) and unprecedented performance (i.e., the first place in the 2015 ImageNet Large Scale Visual Recognition Challenge) at the same time [15]. Residual mapping and shortcut connection are a way of avoiding additional parameters and extra model complexity both by using simpler residual functions instead of their more complicated originals and skipping one or more layers. These methods contributed for the original residual network to achieve a lower error than and eight times as many layers as the Virtual Geometry Group network, the winner of the 2014 ImageNet Large Scale Visual Recognition Challenge, i.e., 3.57% and 152 layers, respectively.

This study modified the original Alex and residual networks by changing the input, output and kernel dimensions from 224x224x3, 1000 (multi-class) and 3 (color image) to 1x2000x1, 2 (binary-class) and 2 (signal), respectively. All ECGs were reviewed manually by two cardiologists in the hospital. This retrospective study got approved by the Institutional Review Board of Korea University Anam Hospital on February 12, 2018 (2018AN0037). Informed consent was waived by the IRB given that data were de-identified. Python 3.6 and Keras 2.2.2 with NVIDIA Titan Xp (12GB RAM) were employed for the analysis on September 2018.

## Results

**Fig. 1:**
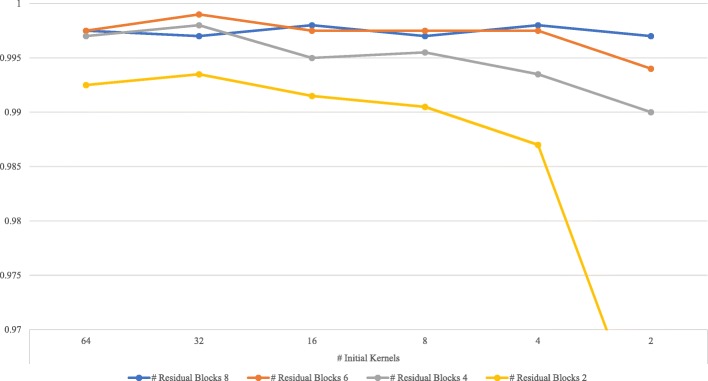
Residual Network: Accuracy over Numbers of Residual Blocks & Initial Kernels

Accuracy measures, epoch numbers and training time for the 30 convolutional networks in this study are displayed in Table 3. In terms of the accuracy, the best network among Alex 1–6 was Alex 3 (0.997) with 24 initial kernels (i.e., kernels in the first layer), 5,268,818 parameters and the training time of 89 s while the best network among Residual 1–1, …, Residual 4–6 was Residual 2–2 (0.999) with 6 residual blocks, 32 initial kernels, 248,418 parameters and the training time of 253 s. It is shown in Fig. 1 how the accuracy of the residual network changes as the numbers of residual blocks and initial kernels change. The performance of the residual network improved as the number of its residual blocks (its depth) increased. The results in Table 3 and Fig. 1 suggest that (1) the number of kernels might not be as significant as that of residual blocks in the case of the residual network and (2) the residual network might be a good model for AF diagnosis with higher accuracy and fewer parameters than its Alex-network counterparts.

**Table 3:** Model Performance: Accuracy, Epoch Number and Training Time

Model	Alex Net 1	Alex Net 2	Alex Net 3	Alex Net 4	Alex Net 5	Alex Net 6
Accuracy	0.9965	0.9960	0.9970^a^	0.9945	0.9950	0.9900
Epoch #	32	61	43	54	51	37
Time (Sec)	163	185	89	110	103	76
Model	Residual 1–1	Residual 1–2	Residual 1–3	Residual 1–4	Residual 1–5	Residual 1–6
Accuracy	0.9975	0.9970	0.9980	0.9970	0.9980	0.9970
Epoch #	62	51	109	56	64	41
Time (Sec)	673	309	440	172	212	162
Model	Residual 2–1	Residual 2–2	Residual 2–3	Residual 2–4	Residual 2–5	Residual 2–6
Accuracy	0.9975	0.9990^b^	0.9975	0.9975	0.9975	0.9940
Epoch #	104	50	41	58	30	28
Time (Sec)	896	253	167	177	93	87
Model	Residual 3–1	Residual 3–2	Residual 3–3	Residual 3–4	Residual 3–5	Residual 3–6
Accuracy	0.9970	0.9980	0.9950	0.9955	0.9935	0.9900
Epoch #	40	55	44	42	39	44
Time (Sec)	322	278	178	129	119	133
Model	Residual 4–1	Residual 4–2	Residual 4–3	Residual 4–4	Residual 4–5	Residual 4–6
Accuracy	0.9925	0.9935	0.9915	0.9905	0.9870	0.9590
Epoch #	26	41	44	43	68	85
Time (Sec)	158	166	134	89	139	173

^a^Best network with the highest accuracy among Alex 1–6

^b^Best network with the highest accuracy among Residual 1–1, …, 4–6

## Discussion

### Main finding of this study

In terms of the accuracy, the best Alex network was one with 24 initial kernels (i.e., kernels in the first layer), 5,268,818 parameters and the training time of 89 s (0.997) while the best residual network was one with 6 residual blocks, 32 initial kernels, 248,418 parameters and the training time of 253 s (0.999). In general, the performance of the residual network improved as the number of its residual blocks (its depth) increased.

### What is already known on this topic

An increasing amount of research has used deep neural networks to diagnose AF and other types of arrhythmia, given their superior performance compared to other machine learning methods. This line of research applied convolutional neural networks (i.e., Alex, Residual), their recurrent counterparts (i.e., long short term memory) or both to achieve the accuracy range of 80–99% with varying numbers of class numbers for ECG data. Most of these studies employed public data such as MIT-BIH arrhythmia data.

### What this study adds

Based on ECG data in a general hospital, this study used more various convolutional neural networks and achieved higher accuracy measures compared to the existing literature for diagnosing arrhythmia as the basis of clinical decision support [6–10]. Specifically, 30 convolutional neural networks were applied and compared for the diagnosis of the NSR vs. AF condition with the accuracy range of 99.00–99.99%: 6 Alex networks with the number of kernels changing from 3 to 256; and 24 residual networks with the number of residuals blocks (or kernels) varying from 8 to 2 (or 64 to 2). Eight cases misspecified by the best residual networks in this study are shown in Additional file 1: Figures S3A - H, e.g., Additional file 1: Figure S3A, AF misspecified as normal by Residual 1–3, 1–4, 3–1 and 3–2. Indeed, six cases specified correctly by all residual networks in this study are shown in Additional file 1: Figures S3I- N. According to these figures, it can be noted that convolutional neural networks find some regular patterns human experts miss. For example, it might be the case in Additional file 1: Figure S3C that (1) the convolutional neural network took four or five beats as a basic unit and predicted the signal as NSR but (2) the human expert considered a single beat as a basic unit and made an opposite diagnosis of AF. It will be an interesting and important topic to understand better how deep neural networks look at signal data and make a diagnosis.

### Limitations of this study

Firstly, this study focused on binary diagnosis of the NSR vs. AF condition. Expanding this study to other arrhythmia conditions might add a great contribution to this line of research. Secondly, comparisons between the convolutional neural networks and their recurrent counterparts in terms of model performance and training time might expand the horizon of research on this topic. Thirdly, a recent review indicates that the standardization of ECG diagnostic criteria is expected to improve the agreement of clinical experts and the performance of computer algorithms regarding ECG interpretation [16]. Much more effort needs to be made in this direction, given that even experienced clinical experts, the gold standard, often disagree in their ECG interpretation. Finally, this study used the training and test sets only. Including the validation set (whose element is not pre-selected into two rhythm types) might be an important next step for advancing science and its clinical practice.

## Conclusions

For AF diagnosis, the residual network might be a good model with higher accuracy and fewer parameters than its Alex-network counterparts.

## Supplementary information

**Additional file 1:** **Figure S1.** Electrocardiogram Wave. **A** Normal. **B** Atrial Fibrillation vs. Normal. The atrial-fibrillation rhythm in the top does not have a P wave (purple arrow) of the normal rhythm in the bottom. **Figure S2.** Preprocessing. **A**. Removing the Background Grid. **B**. Selecting Target Signals. **C**. Getting Numeric Values. **Figure S3. A**. AF Misspecified as Normal by Residual 1–3, 1–4, 3–1 and 3–2 (1/3). **B**. AF Misspecified as Normal by Residual 1–1, 1–2, 1–3, 1–4, 1–6 and 2–5 (2/3). **C**. AF Misspecified as Normal by Residual 1–1, 1–2, 1–3, 1–4, 1–5, 1–6, 2–1, 2–2 and 3–2 (3/3). **D**. Normal Misspecified as AF by Residual 1–1, 1–2, 1–6, 2–3 and 2–4 (1/5). **E**. Normal Misspecified as AF by Residual 1–1, 1–2, 1–5, 2–1, 2–2, 2–3, 2–4, 2–5 and 3–1 (2/5). **F.** Normal Misspecified as AF by Residual 1–1, 1–2, 1–3, 1–5, 2–1, 2–3, 2–4, 2–5, 3–1 and 3–2 (3/5). **G**. Normal Misspecified as AF by Residual 1–2, 1–5, 2–1, 2–3, 2–5 and 3–1 (4/5). **H**. Normal Misspecified as AF by Residual 1–4, 2–1, 2–3, 2–4, 2–5 and 3–1 (5/5). **I**. Normal Specified as Normal by Residual Networks (1/3). **J.** Normal Specified as Normal by Residual Networks (2/3). **K.** Normal Specified as Normal by Residual Networks (3/3). **L.** AF Specified as AF by Residual Networks (1/3). **M**. AF Specified as AF by Residual Networks (2/3). **N**. AF Specified as AF by Residual Networks (3/3).

## Data Availability

The datasets used and/or analysed during the current study are available from the corresponding author on reasonable request.

## Abbreviations

*AF*: Atrial fibrillation
*ECG*: Electrocardiogram
*MIT-BIH*: Massachusetts Institute of Technology-Beth Israel Hospital
*NSR*: Normal sinus rhythm
