# Supplementary material for: Atrial fibrillation classification based on convolutional neural networks
Source: BMC Med Inform Decis Mak. 2019 Oct 29;19:206. doi: 10.1186/s12911-019-0946-1 (PMC6819477; doi:10.1186/s12911-019-0946-1)

Figure S1. Electrocardiogram Wave

1. Normal


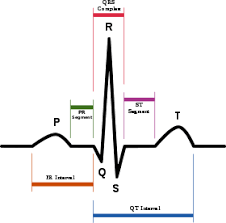


Source: Wikipedia

1. Atrial Fibrillation vs. Normal


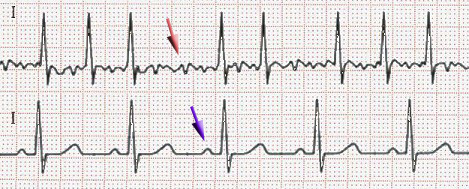


The atrial-fibrillation rhythm in the top does not have a P wave (purple arrow) of the normal rhythm in the bottom

Source: Wikipedia

Figure S2. Preprocessing

1. Removing the Background Grid


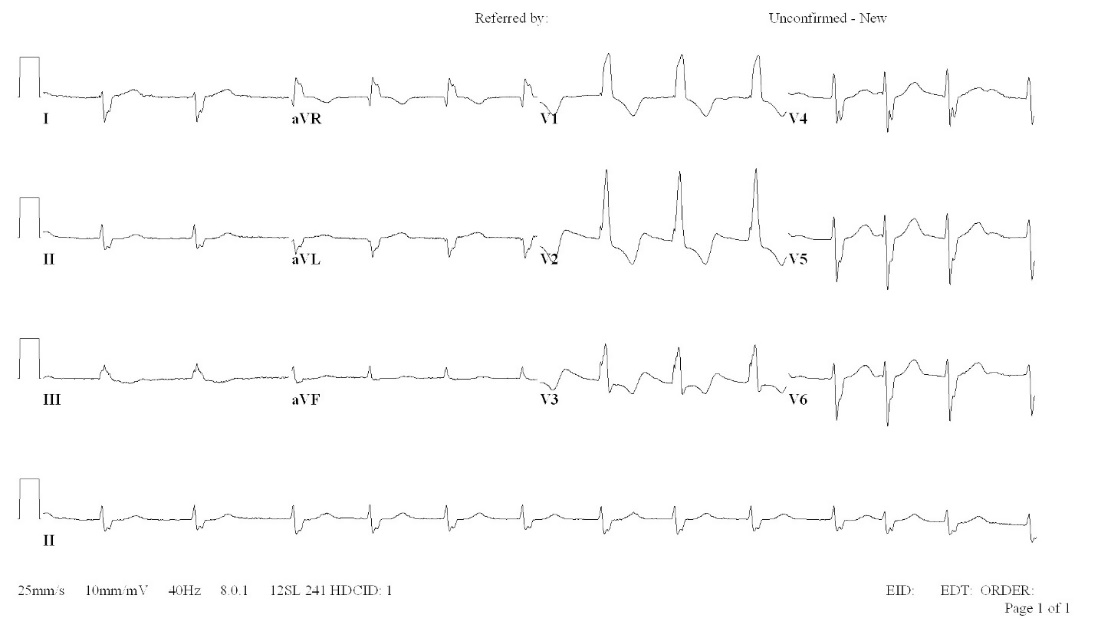


1. Selecting Target Signals


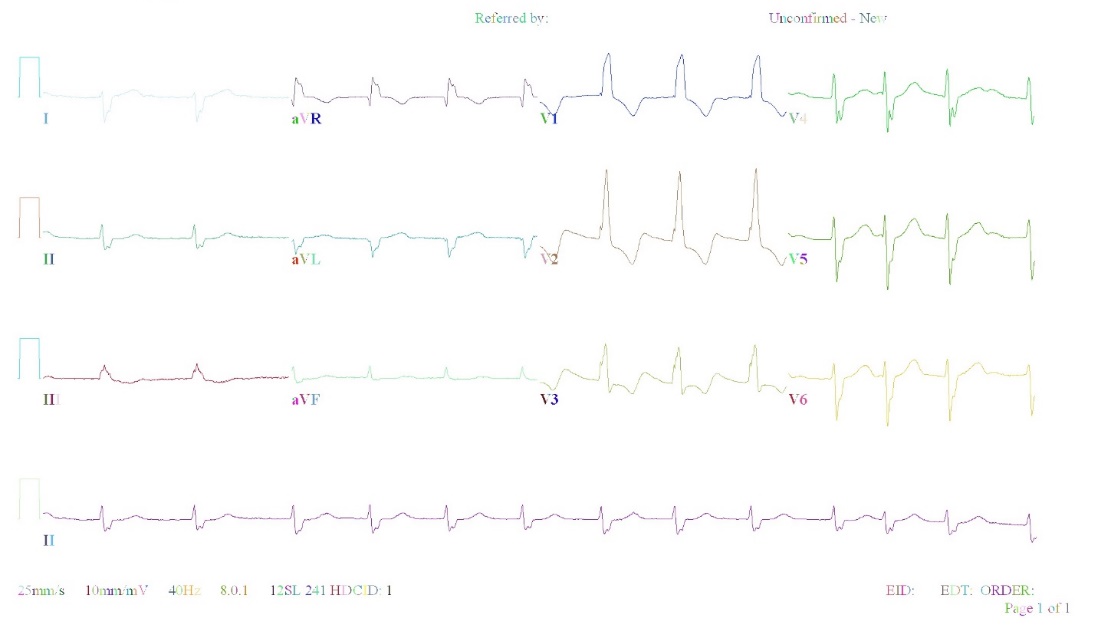


1. Getting Numeric Values


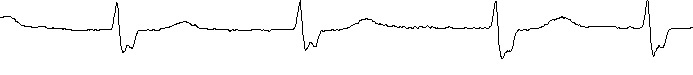


…

time

mV

Figure S3.

1. AF Misspecified as Normal by Residual 1-3, 1-4, 3-1 and 3-2 (1/3)


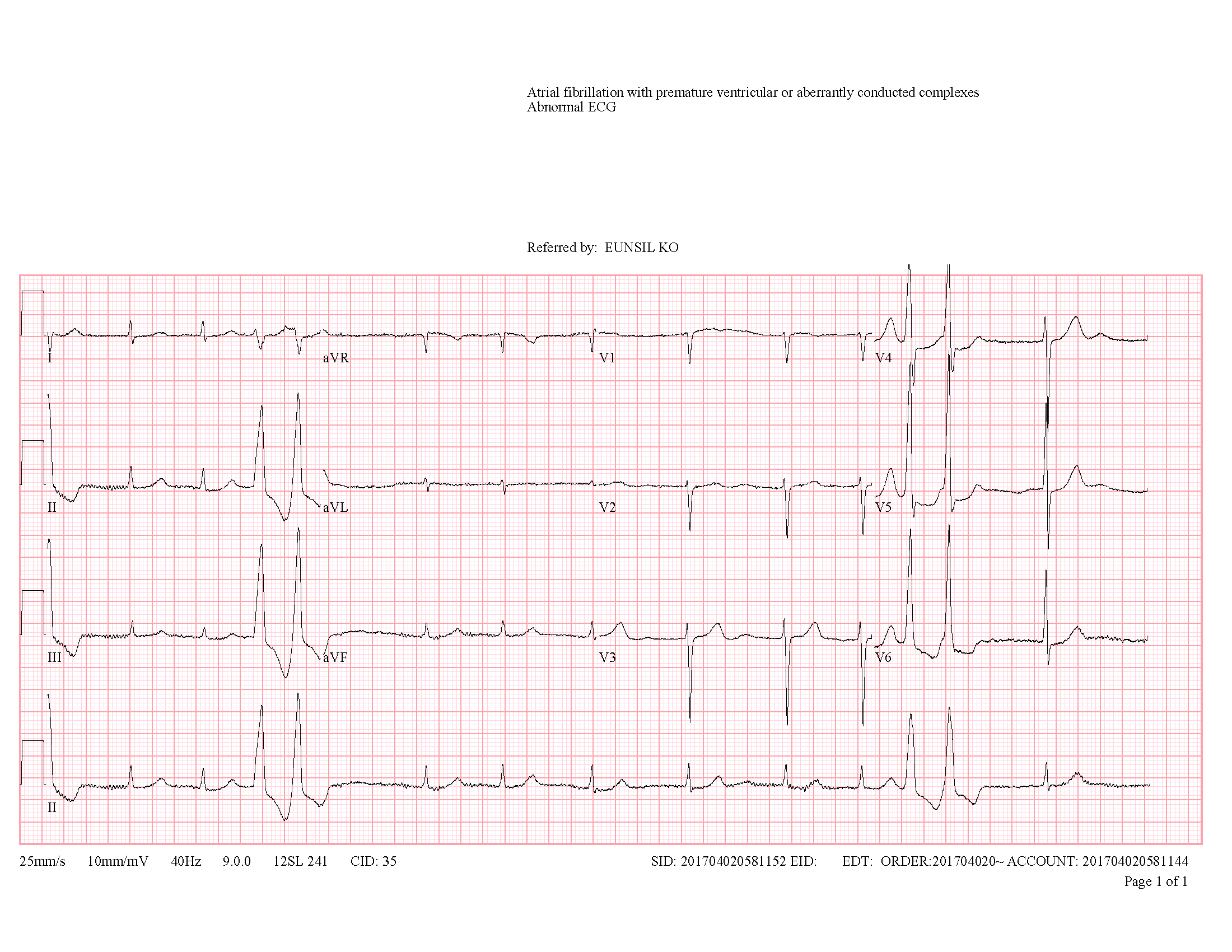


1. AF Misspecified as Normal by Residual 1-1, 1-2, 1-3, 1-4, 1-6 and 2-5 (2/3)


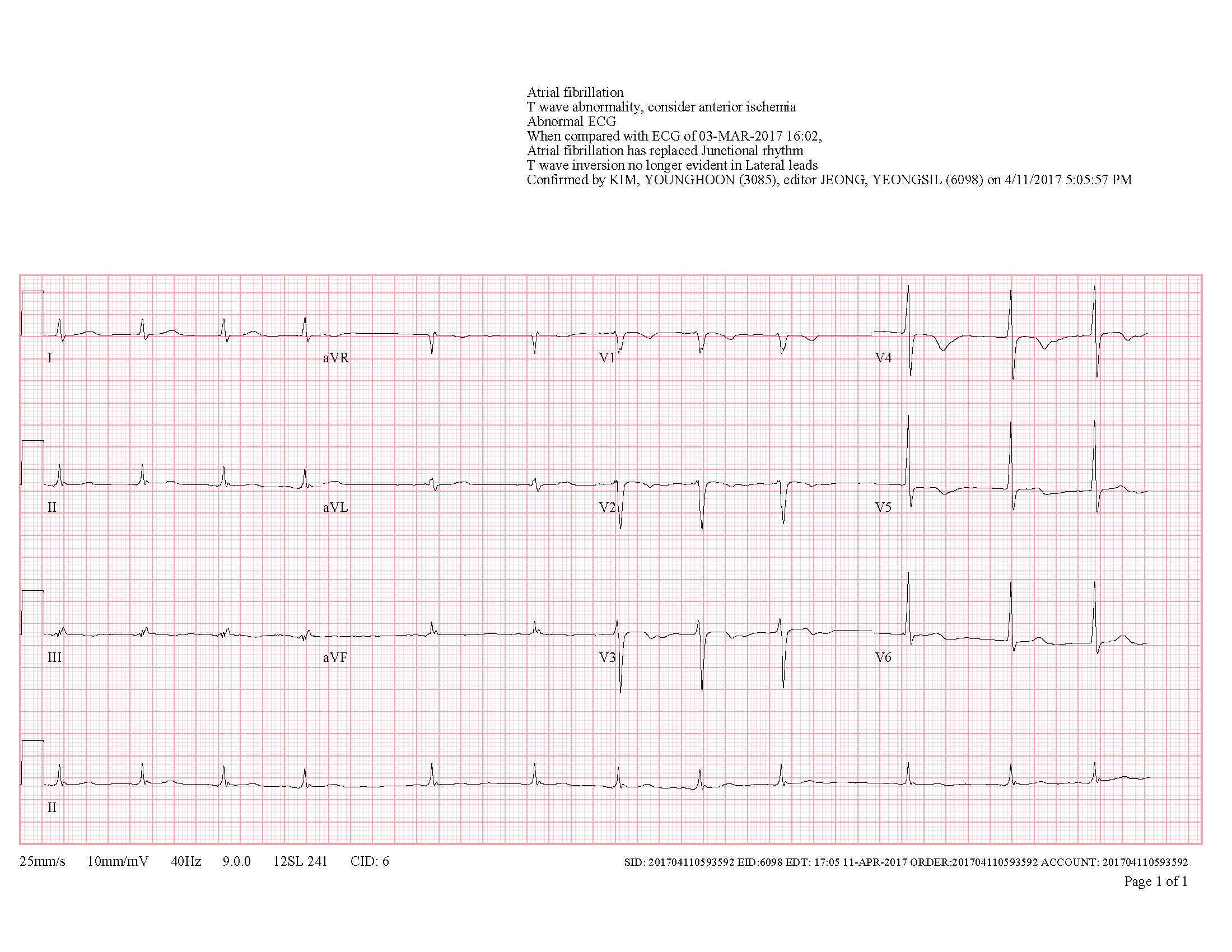


1. AF Misspecified as Normal by Residual 1-1, 1-2, 1-3, 1-4, 1-5, 1-6, 2-1, 2-2 and 3-2 (3/3)


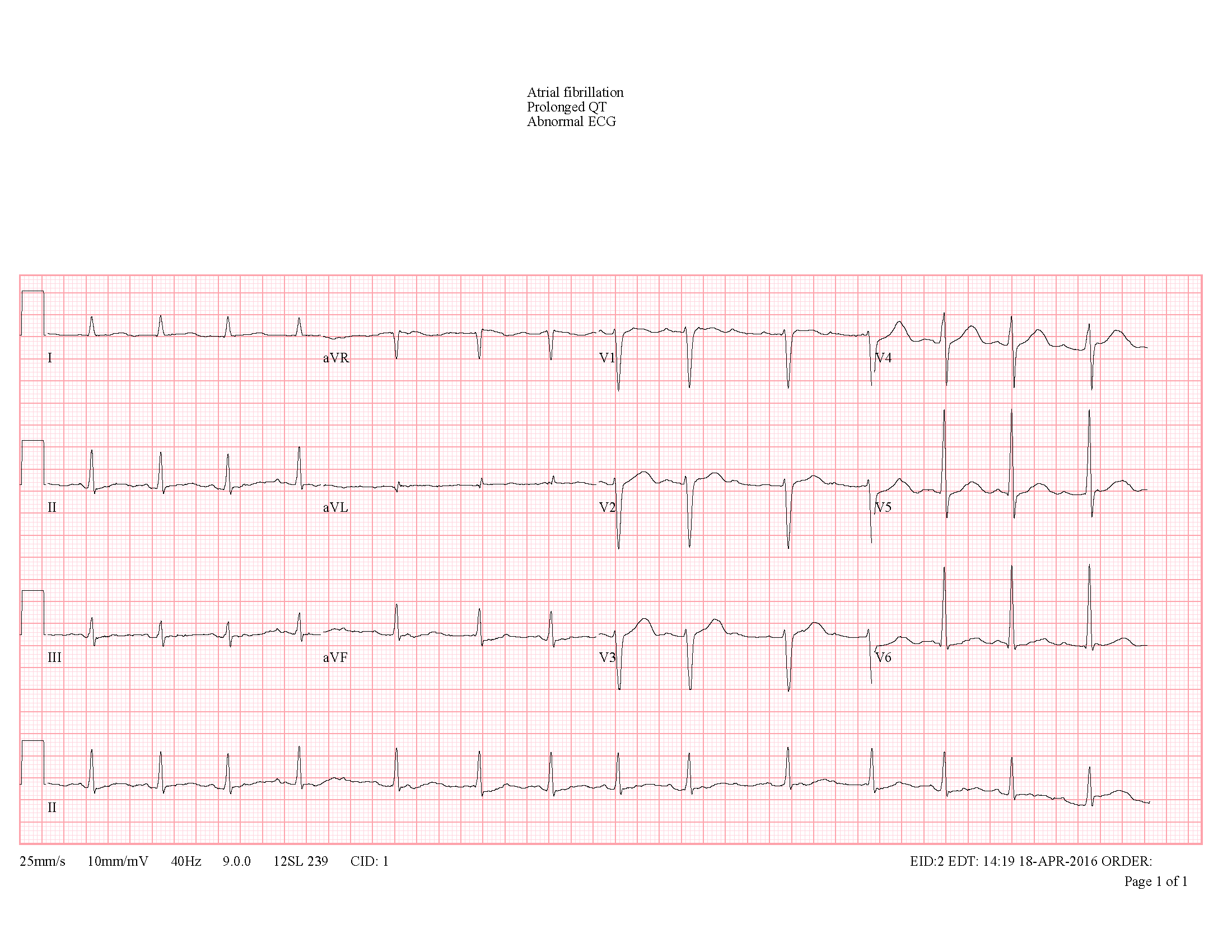


1. Normal Misspecified as AF by Residual 1-1, 1-2, 1-6, 2-3 and 2-4 (1/5)


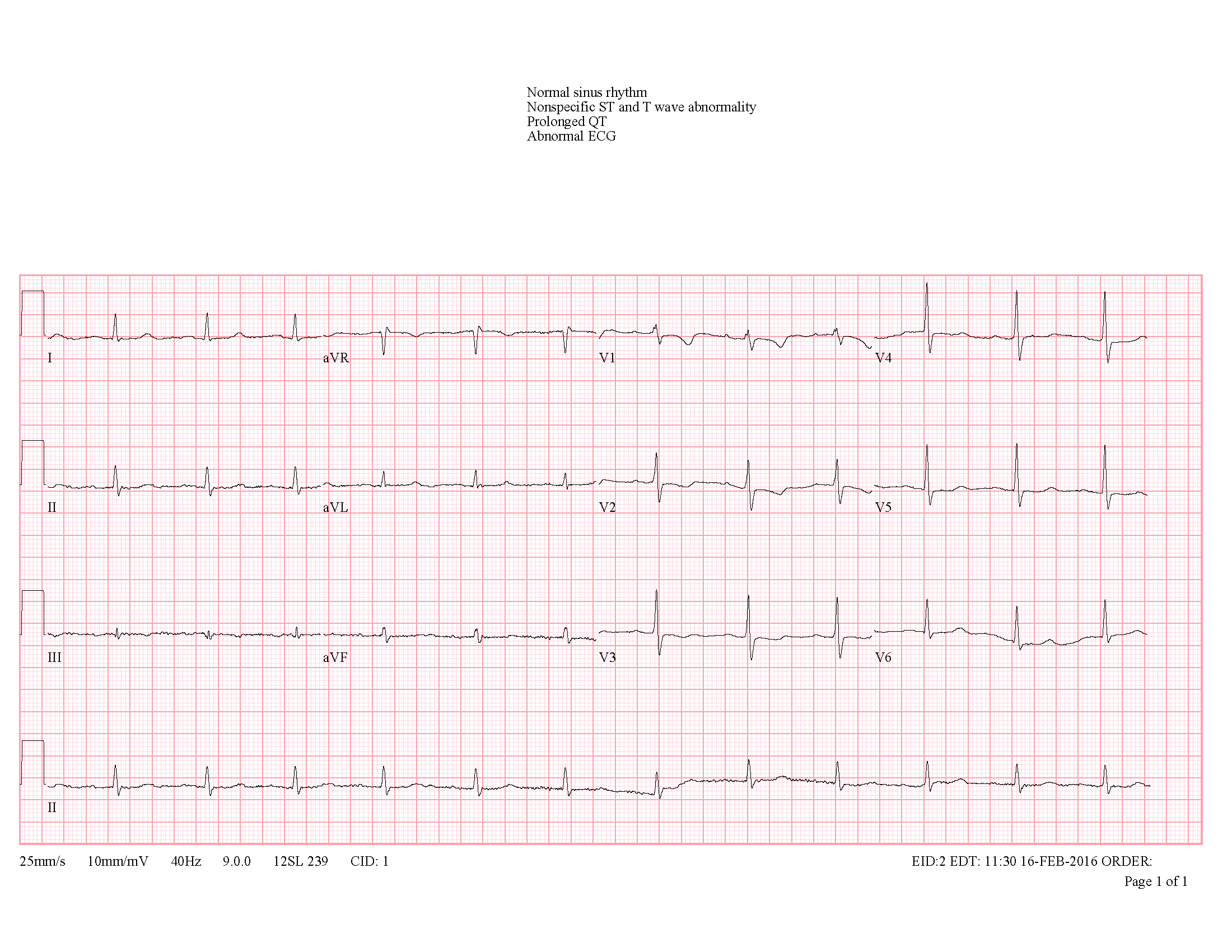


1. Normal Misspecified as AF by Residual 1-1, 1-2, 1-5, 2-1, 2-2, 2-3, 2-4, 2-5 and 3-1 (2/5)


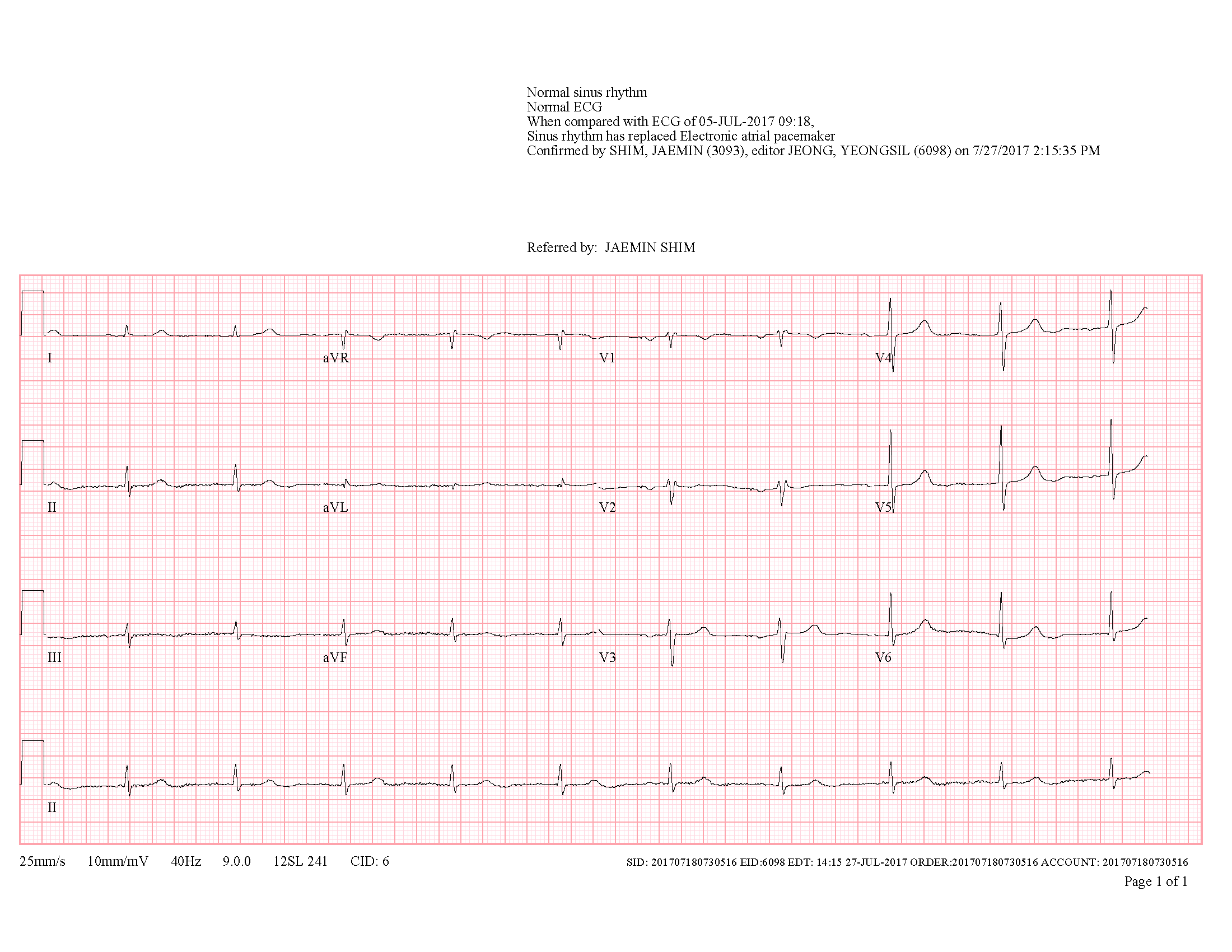


1. Normal Misspecified as AF by Residual 1-1, 1-2, 1-3, 1-5, 2-1, 2-3, 2-4, 2-5, 3-1 and 3-2 (3/5)


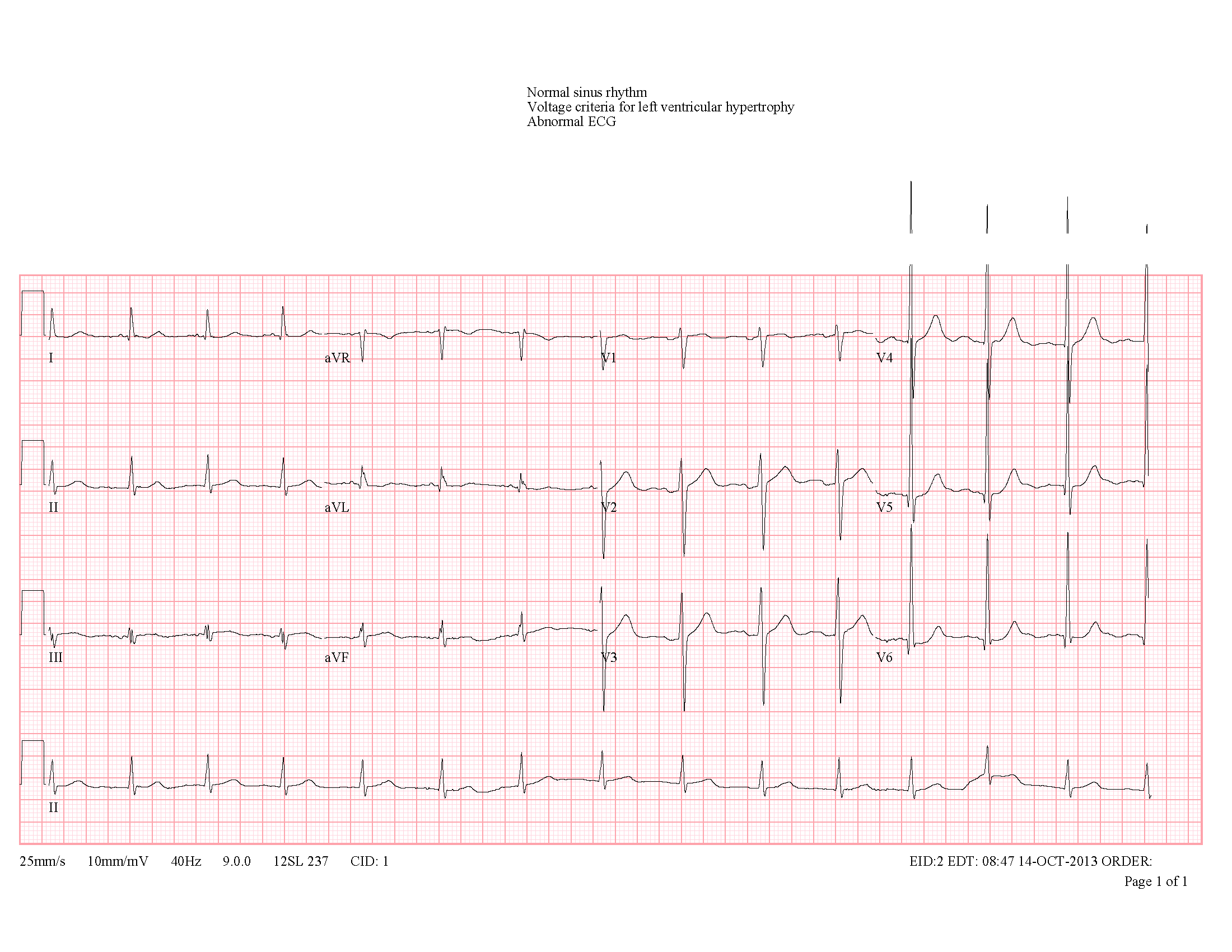


1. Normal Misspecified as AF by Residual 1-2, 1-5, 2-1, 2-3, 2-5 and 3-1 (4/5)


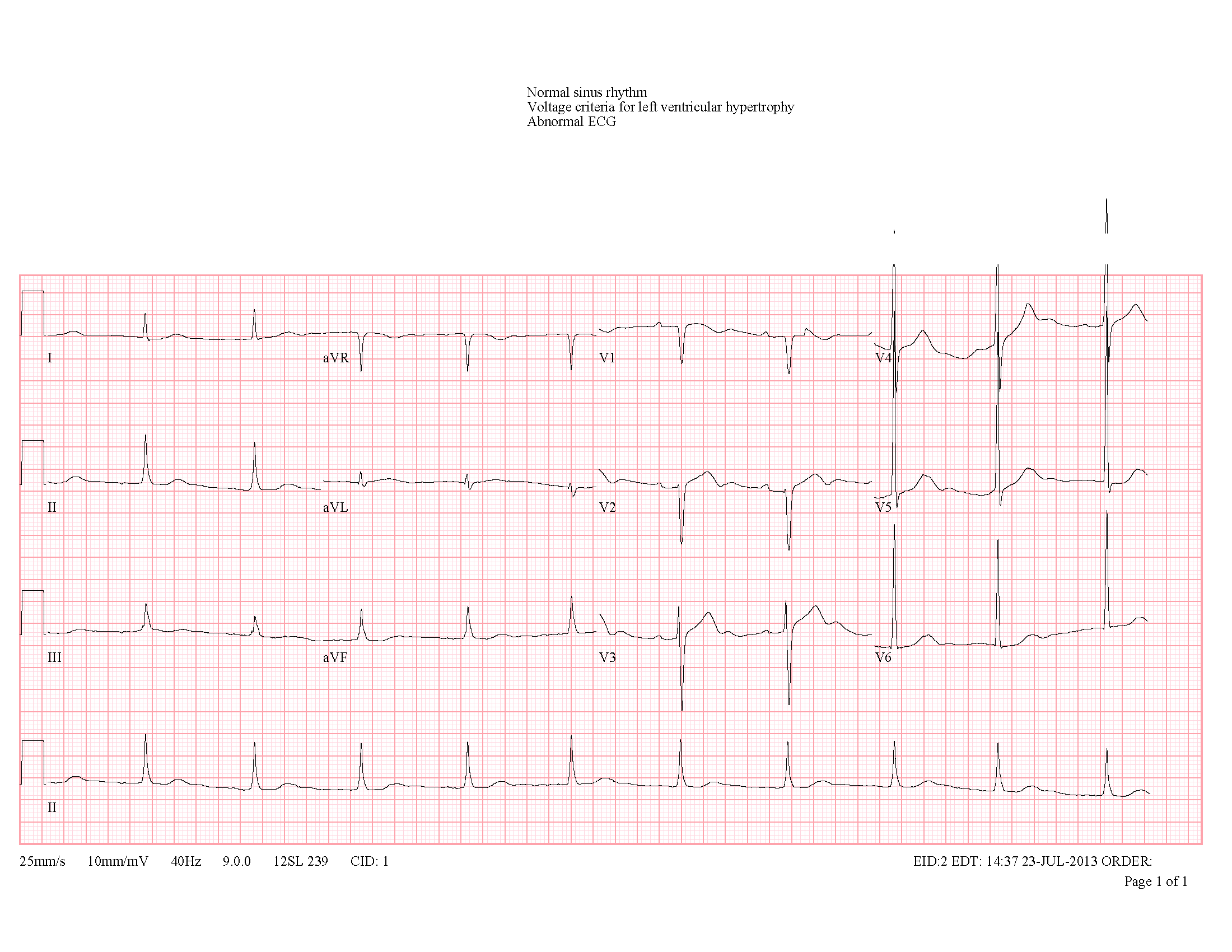


1. Normal Misspecified as AF by Residual 1-4, 2-1, 2-3, 2-4, 2-5 and 3-1 (5/5)


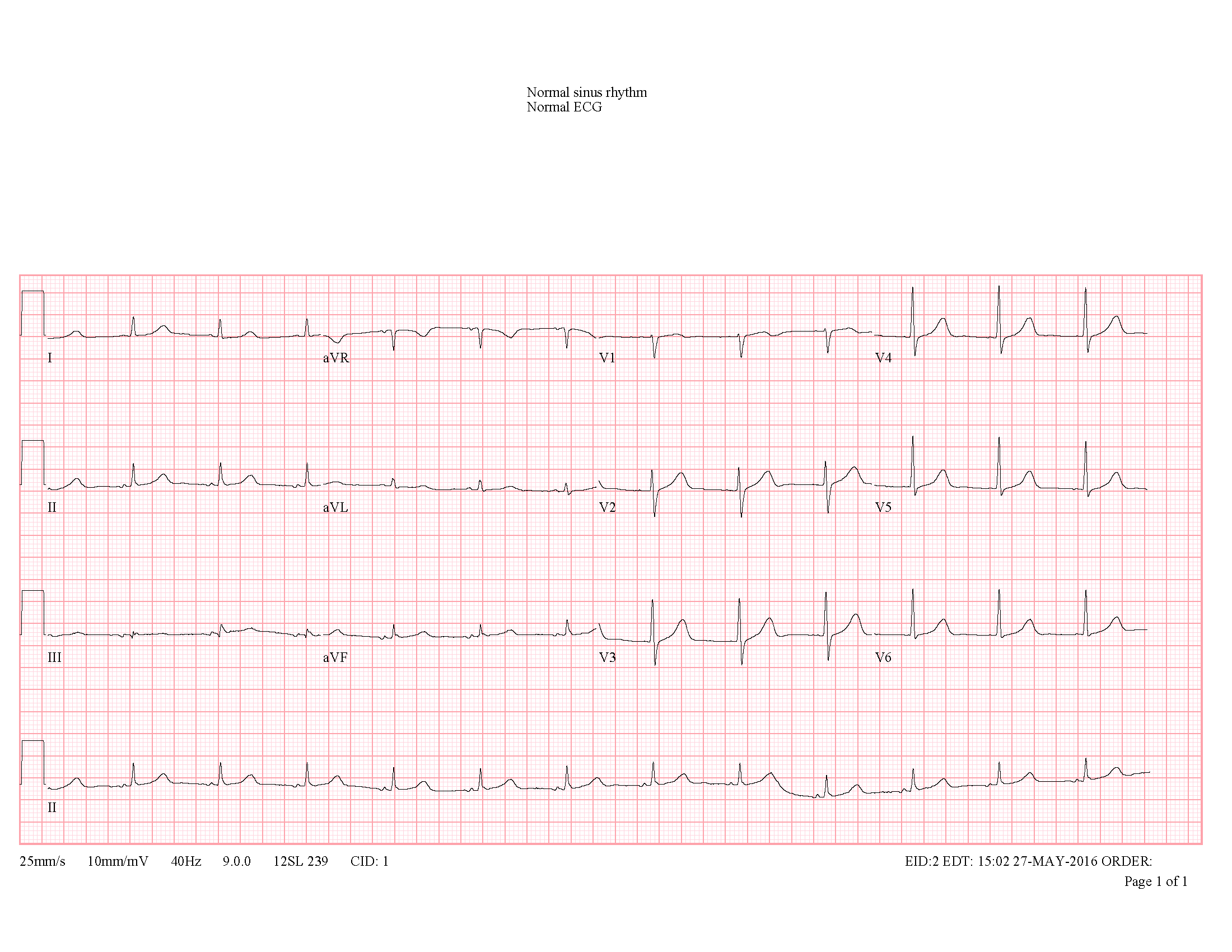


1. Normal Specified as Normal by Residual Networks (1/3)


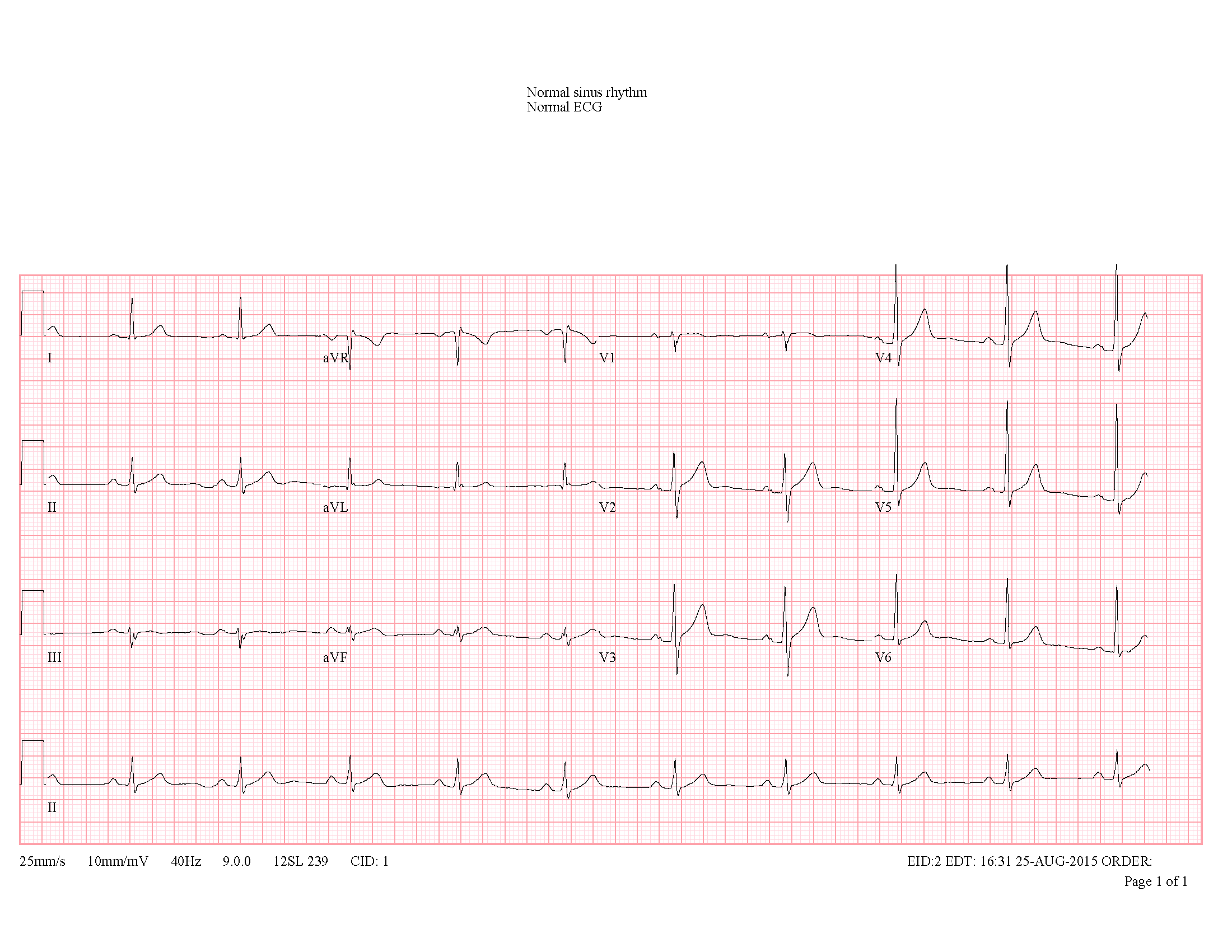


1. Normal Specified as Normal by Residual Networks (2/3)


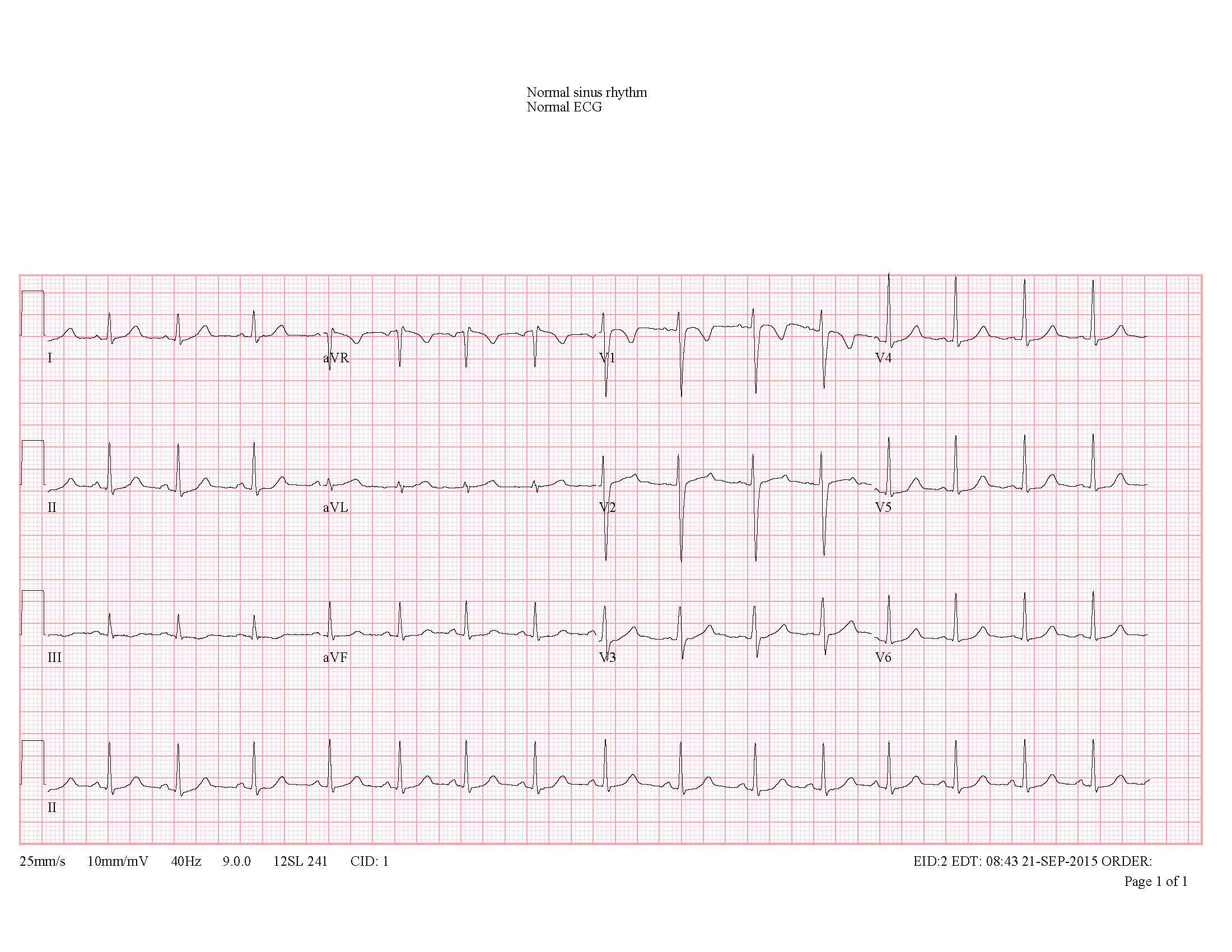


1. Normal Specified as Normal by Residual Networks (3/3)


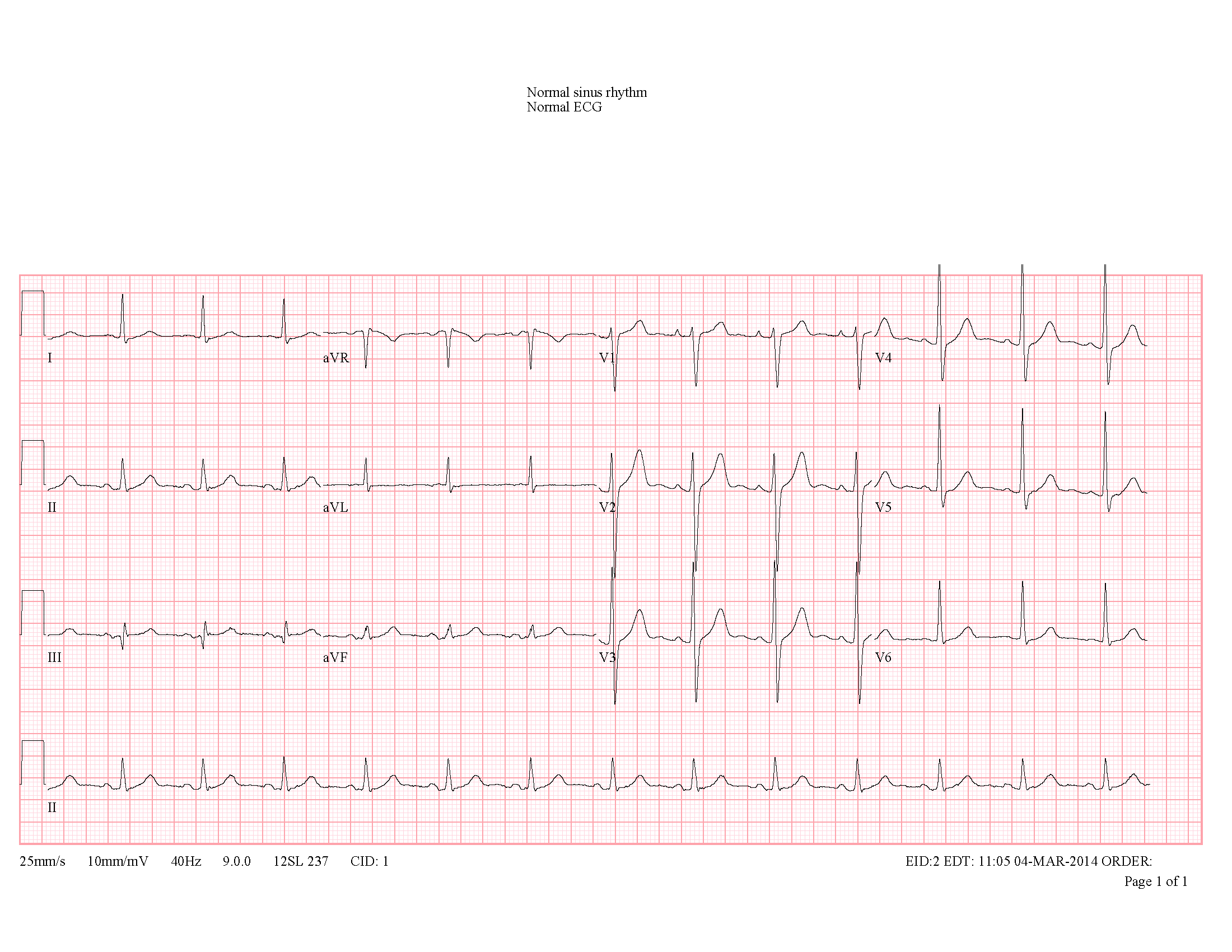


1. AF Specified as AF by Residual Networks (1/3)


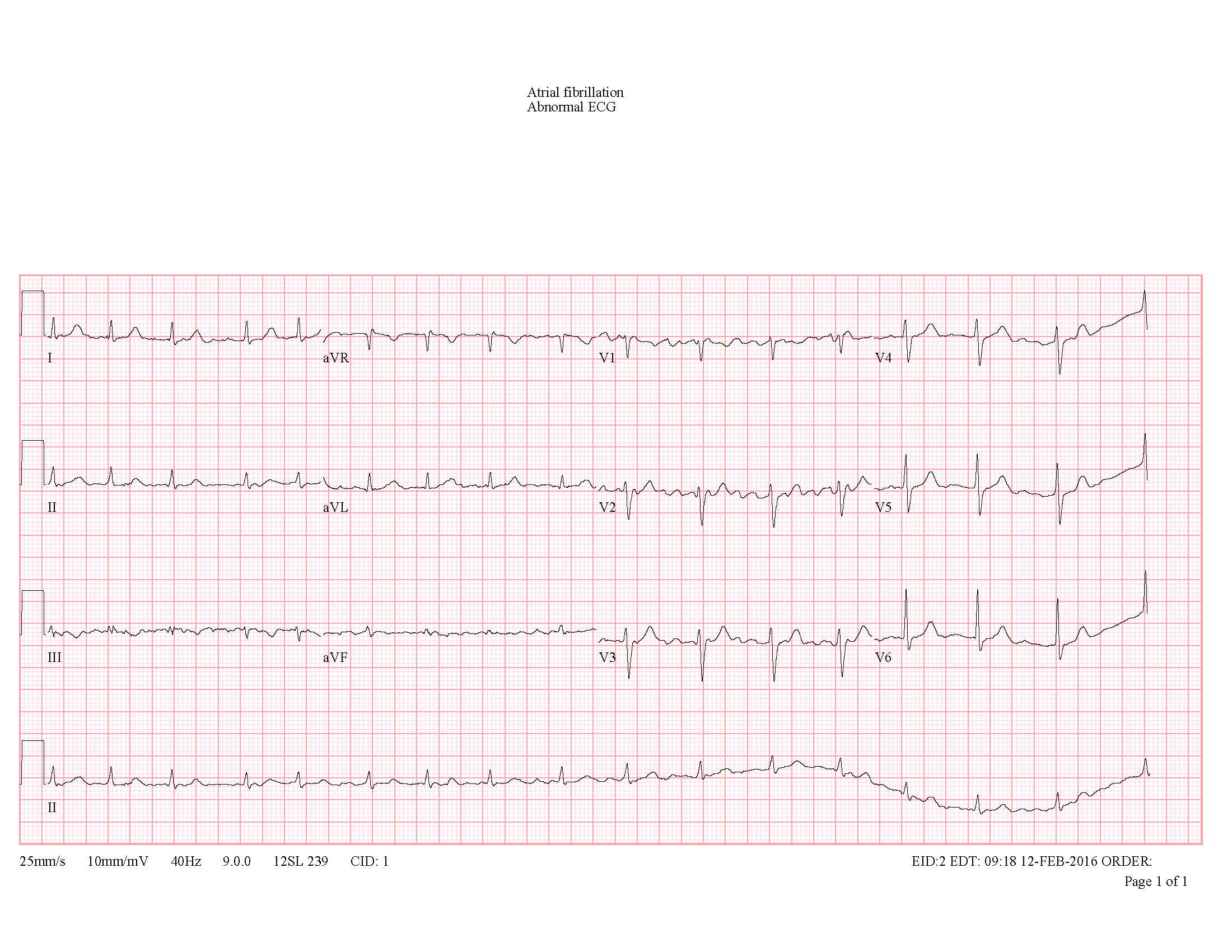


1. AF Specified as AF by Residual Networks (2/3)


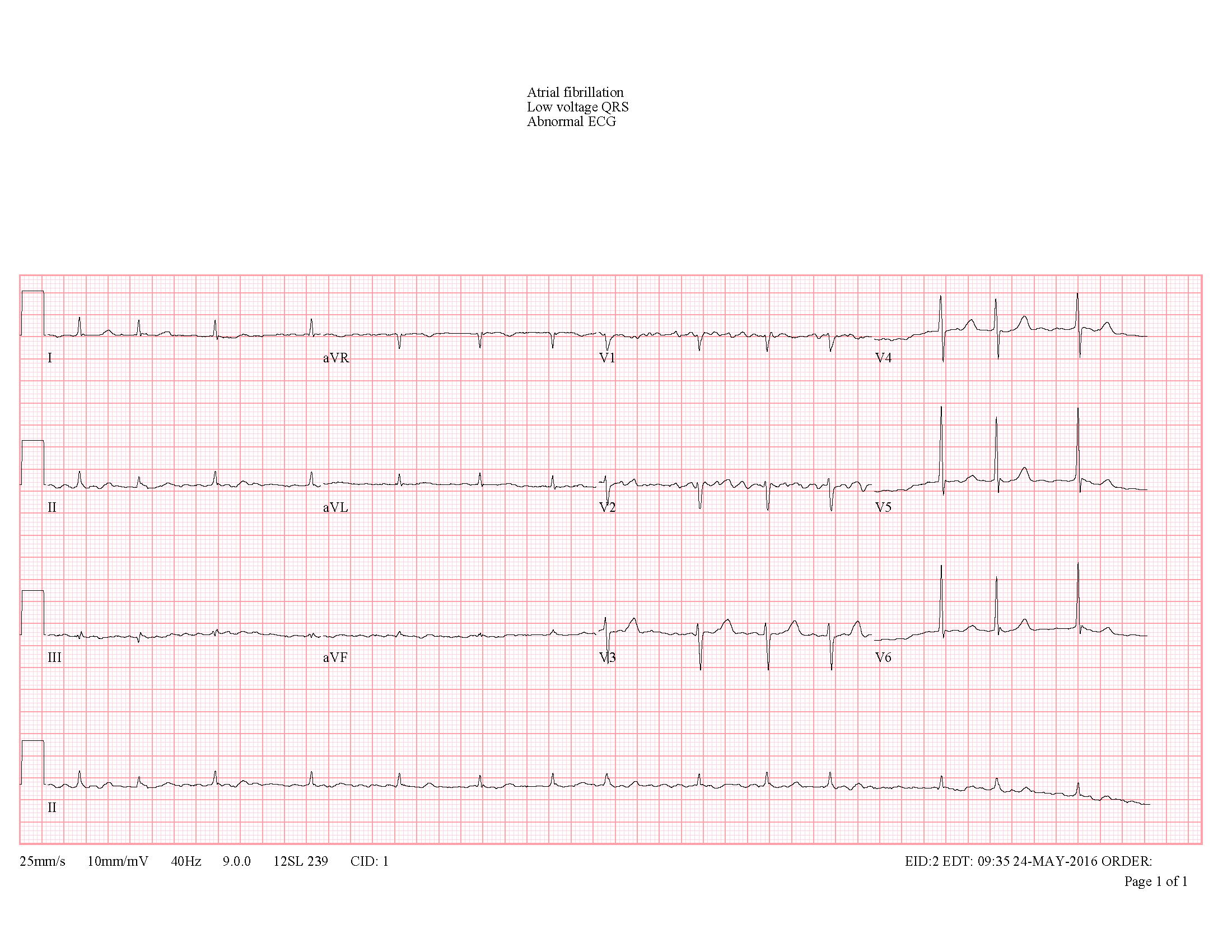


1. AF Specified as AF by Residual Networks (3/3)


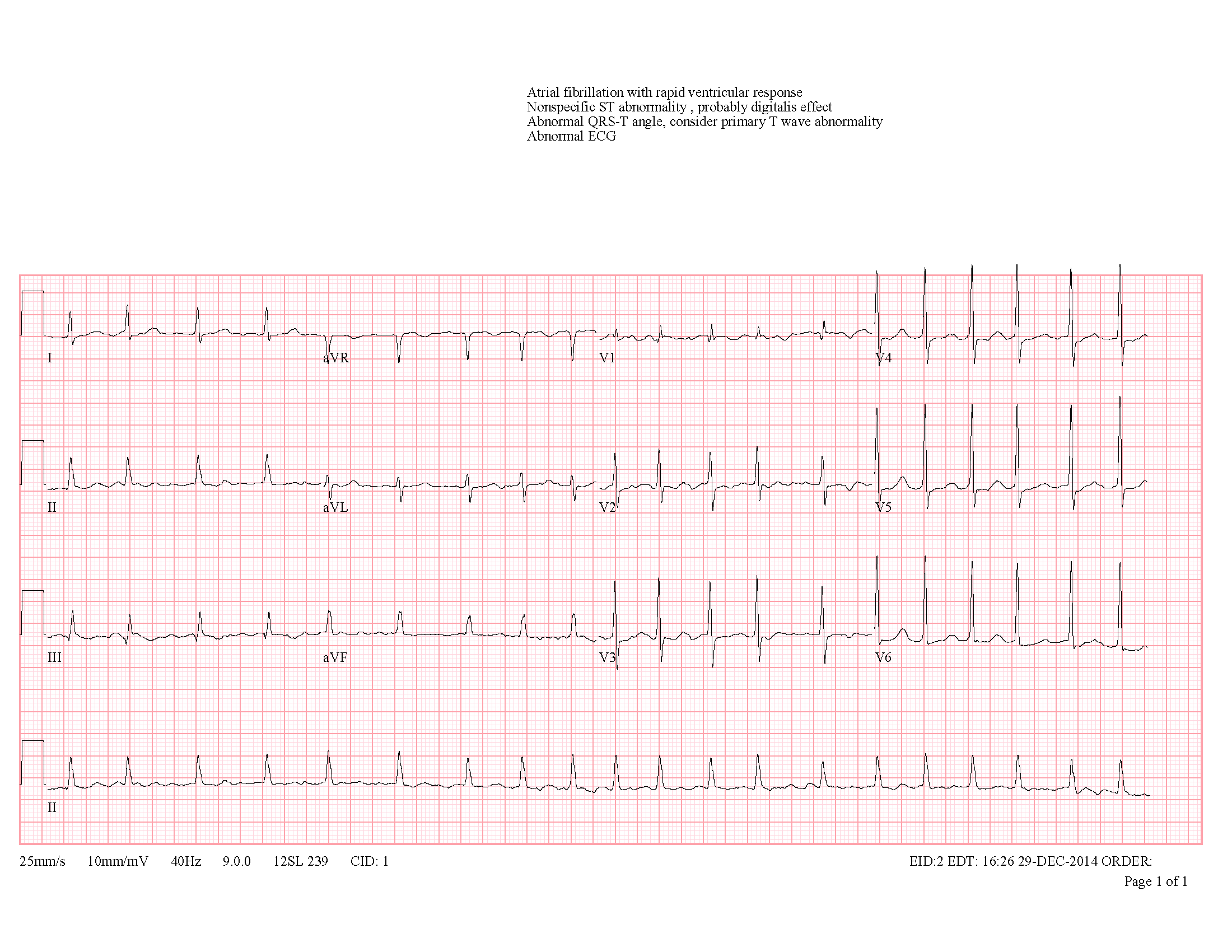

Supplement: Supplementary file 1 — Additional file 1: Figure S1. Electrocardiogram Wave. A Normal. B Atrial Fibrillation vs. Normal. The atrial-fibrillation rhythm in the top does not have a P wave (purple arrow) of the normal rhythm in the bottom. Figure S2. Preprocessing. A. Removing the Background Grid. B. Selecting Target Signals. C. Getting Numeric Values. Figure S3. A. AF Misspecified as Normal by Residual 1–3, 1–4, 3–1 and 3–2 (1/3). B. AF Misspecified as Normal by Residual 1–1, 1–2, 1–3, 1–4, 1–6 and 2–5 (2/3). C. AF Misspecified as Normal by Residual 1–1, 1–2, 1–3, 1–4, 1–5, 1–6, 2–1, 2–2 and 3–2 (3/3). D. Normal Misspecified as AF by Residual 1–1, 1–2, 1–6, 2–3 and 2–4 (1/5). E. Normal Misspecified as AF by Residual 1–1, 1–2, 1–5, 2–1, 2–2, 2–3, 2–4, 2–5 and 3–1 (2/5). F. Normal Misspecified as AF by Residual 1–1, 1–2, 1–3, 1–5, 2–1, 2–3, 2–4, 2–5, 3–1 and 3–2 (3/5). G. Normal Misspecified as AF by Residual 1–2, 1–5, 2–1, 2–3, 2–5 and 3–1 (4/5). H. Normal Misspecified as AF by Residual 1–4, 2–1, 2–3, 2–4, 2–5 and 3–1 (5/5). I. Normal Specified as Normal by Residual Networks (1/3). J. Normal Specified as Normal by Residual Networks (2/3). K. Normal Specified as Normal by Residual Networks (3/3). L. AF Specified as AF by Residual Networks (1/3). M. AF Specified as AF by Residual Networks (2/3). N. AF Specified as AF by Residual Networks (3/3). [file 12911_2019_946_MOESM1_ESM.doc]
